# Sensory processing sensitivity, anxiety, and short-term heart rate variability in older adults living at high southern latitudes: a brief report

**DOI:** 10.3389/fpsyt.2025.1736196

**Published:** 2026-01-12

**Authors:** Diego Baeza, Leyla Huirimilla-Casanova, Claudia Estrada, Salvador Buccella, Matías Castillo-Aguilar, Cristian Núñez-Espinosa

**Affiliations:** 1 Centro Asistencial Docente e Investigación (CADI UMAG), Punta Arenas, Chile; 2 Departamento de Psicología, Universidad de Magallanes (UMAG), Punta Arenas, Chile; 3 Escuela de Medicina, Universidad de Magallanes (UMAG), Punta Arenas, Chile

**Keywords:** anxiety, heart rate variability, high sensitivity, older adults, sensory processing sensitivity

## Abstract

**Background:**

Populations living at high southern latitudes are under-represented in aging and psychophysiology research, despite distinctive environmental stressors (long winters, marked seasonality, isolation). Objectives: To test associations between SPS, anxiety, and HRV in community-dwelling older adults living at high southern latitudes.

**Methods:**

We enrolled 101 older adults (mean age 71 years; 72% women) from CADI-UMAG. SPS was measured with the 27-item Highly Sensitive Person Scale (HSPS) and anxiety with the Beck Anxiety Inventory (BAI, clinical cut-off ≥16). HRV (5-min artifact-free) was recorded at rest and after a 2-min step/knee-raise test. Bayesian hierarchical models (medians, 95% CrI, pd, ROPE, BF10) accounted for within-subject correlation and seasonality.

**Results:**

HSPS was positively associated with anxiety: a 1-SD increase in HSPS corresponded to a 0.422-SD increase in BAI. Seasonality showed strong evidence for a null effect (BF10 = 0.08). BAI showed no meaningful associations with resting HRV indices—RMSSD (BF10 = 0.046), SDNN (0.200), HF (0.070), LF (0.032), VLF (0.038)—and HSPS did not moderate BAI–HRV links nor HRV responses to exercise (e.g., ΔRMSSD–BAI median 0.003; ROPE = 100%).

**Conclusions:**

In older adults living at high southern latitudes, SPS appears to be associated with anxiety but not to conventional short-term HRV markers, suggesting SPS may reflects psychological vulnerability rather than parasympathetic dysfunction detectable with brief HRV recordings. These findings highlight the need for context-aware mental-health strategies for highly sensitive older adults in understudied southern populations.

## 1 Introduction

Older adults living at high southern latitudes are exposed to unique environmental patterns (extensive light seasonality, intense wind, low temperature, and increased isolation) that may modulate environmental sensitivity, emotional regulation, and autonomic circuitry (1–3). However, this geographic and demographic group is markedly underrepresented in the psychophysiology of aging literature. In this context, Sensory Processing Sensitivity (SPS), a trait that describes heightened detection and processing of environmental subtleties, may interact with anxiety and heart rate variability (HRV), a biomarker of autonomic control, in ways specific to these extreme southern conditions.

Research in the field of psychology has highlighted the importance of understanding how biological and psychological factors interact in the overall well-being of human beings (4, 5). Each person perceives and integrates environmental stimuli in a particular way, which translates into responses of greater or lesser intensity compared to other individuals (6, 7).

SPS is recognized as a temperamental and phenotypic trait (8) characterized by a tendency to a deeper processing of sensory information, increased emotional reactivity and empathy and heightened awareness of environmental subtleties, often accompanied by ease of overstimulation. This characteristic often translates into an intense emotional response and greater empathy toward the affective signals of others (9). Far from being an isolated phenomenon, SPS reflects individual differences in sensitivity to environmental stimuli and constitutes a common trait, with a hereditary basis and preserved throughout evolution (10). Early studies estimate that approximately 15 -20% of the population scores high on the SPS trait, suggesting an evolutionary conserved minority phenotype (7).

Individuals with SPS tend to experience heightened arousal when exposed to various stimuli, although this response can be attenuated by states of physiological calm and homeostasis mechanisms, which promote better cognitive and emotional control (8). This interindividual variability appears to be modulated by factors such as temperament, physiological reactivity, and developmental plasticity (7). Available evidence also suggests that high sensitivity may be associated with greater vulnerability to depression and anxiety (11), a finding that deserves attention in clinical practice, especially in vulnerable populations such as older adults.

Among the changes in an individual’s subjective state is anxiety as an emotional response to perceived threats (5). This state affects cognitive flexibility, i.e., the ability to adapt to new demands and switch between strategies, which is often compromised in anxious individuals (12). Anxiety has been linked to characteristic cognitive biases, such as the tendency to interpret ambiguous or neutral situations as threatening and to respond with disproportionate fear (13).

In this context, it has been documented those difficulties associated with SPS during childhood can predict the onset of anxiety disorders in adulthood, with emotional dysregulation being a key factor in this trajectory (14) (15). These observations suggest an early link between sensitivity, emotional regulation, and anxiety risk. Although research on sensory processing difficulties in young people with mental or neurodevelopmental conditions is still limited, most studies agree that these challenges are closely linked to such disorders. This evidence suggests that being unusually sensitive or reactive to sensory experiences may be a shared characteristic across different conditions, one that can contribute to emotional difficulties and increase vulnerability to anxiety or obsessive-compulsive symptoms during childhood and adolescence (16).

Heart rate variability (HRV) has established itself as a reliable biomarker of autonomic activity, particularly parasympathetic tone (17, 18). It is measured through the variation in R-R intervals on an electrocardiogram, reflecting the dynamic interaction between the autonomic nervous system and cardiac control (17, 19). There are different methods of analysis: in the time domain, frequency domain, nonlinear approaches, and others that integrate the respiratory signal. Among the most widely used indices, high-frequency (HF) power, root mean square of successive differences (RMSSD), and respiratory sinus arrhythmia (RSA) are considered parasympathetic markers, while the standard deviation of normal-to-normal intervals (SDNN) and total power (TP) reflect both sympathetic and parasympathetic components (17, 18).

In clinical terms, higher HRV usually indicates a flexible and efficient physiological regulation system, while reduced values have been associated with autonomic dysfunctions and various psychological disorders (13). It is no coincidence that people with anxiety disorders have lower HRV than individuals without such symptoms (17, 19).

SPS is associated with greater emotional reactivity and, in some studies, with greater vulnerability to anxiety. HRV, particularly RMSSD and HF, captures short-term vagal modulation (20). However, evidence linking SPS-anxiety-HRV in older adults, and especially in southern populations, is scarce.

This study aims to relate Sensory Processing Sensitivity, Anxiety, and Short-Term Heart Rate Variability in Older Adults Living at High Southern Latitudes.

## 2 Materials and methods

### 2.1 Study design

An observational, descriptive–correlational study with a quantitative approach was conducted, in which Sensory Processing Sensitivity, anxiety symptoms, and heart rate variability (HRV) were assessed at two time points while modeling the intra-subject correlation. Seasonality was operationalized through two assessment periods corresponding to late winter and late summer at high southern latitudes, and included as a predictor in the hierarchical models to account for within-participant seasonal variation.

### 2.2 Participants

The study was carried out in the extreme south of Chile (CADI-UMAG), a high southern latitude environment with marked seasonality. The convenience sample consisted of 101 participants, ranging in age from 60 to 89 years, with a mean age of 71 years. Seventy-two percent (n=73) of the participants were women and 28% (n=28) were men (see **Table 1**). Most participants had completed at least secondary education. All participants signed the informed consent form, which informed them of their rights as part of the study.

**Table 1:** Effect of BAI and HSPS scores on HRV measurements, adjusted for the effect of seasonality and intra-subject correlations. RMSSD (Root mean squared of successive differences between N-N intervals); SDNN (Standard Deviation of NN Intervals) HF (High Frequency); LF (Low Frequency); VLF (Very Low Frequency); PNSN (Parasympathetic Nervous System Index); SNSN (Sympathetic Nervous System Index).

PARÁMETRO		MEDIANA	BAJO	ALTO	PD	PS	ROPE	BF
RMSSD	Pre BAI (Score)	0.065	-0.107	0.225	0.773	0.341	0.666	0.039
	Pre HSPS (Score).	0.043	-0.123	0.216	0.687	0.251	0.740	0.032
SDNN	Pre BAI (Score)	0.113	-0.048	0.279	0.906	0.563	0.433	0.072
	Pre HSPS (Score)	0.089	-0.077	0.249	0.852	0.447	0.556	0.052
HF	Pre BAI (Score)	0.121	-0.052	0.288	0.919	0.600	0.395	0.074
	Pre BAI (Score)	-0.035	-0.204	0.134	0.655	0.220	0.756	0.033
LF	Pre BAI (Score)	-0.034	-0.202	0.141	0.650	0.219	0.754	0.032
	Pre HSPS (Score)	0.182	0.014	0.357	0.982	0.823	0.160	0.262
VLF	Pre BAI (Score)	0.018	-0.156	0.193	0.581	0.185	0.763	0.031
	Pre HSPS (Score)	0.109	-0.67	0.280	0.886	0.539	0.459	0.063
PNSN	Pre BAI (Score)	0.046	-0.128	0.215	0.703	0.274	0.713	0.034
	Pre HSPS (Score)	-0.017	-0.172	0.172	0.570	0.167	0.770	0.031
SNSN	Pre BAI (Score)	-0.125	-0.292	0.042	0.925	0.613	0.381	0.081
	Pre HSPS (Score)	-0.016	-0.180	0.153	0.573	0.163	0.790	0.29

Participants with pacemakers, diagnosed dementia, motor disabilities precluding testing, or those using stimulants in the 12 hours prior to cardiac measurement were excluded. Furthermore, chronic use of medications with a known impact on HRV (e.g., beta-blockers, antidepressants, benzodiazepines, anticholinergics) was systematically recorded so that it could be included as a covariate in sensitivity analyses.

### 2.3 Instruments

#### 2.3.1 Beck anxiety inventory

Assesses the presence of anxiety symptoms using 21 self-administered items, each of which presents a sign or symptom. The assesses must choose from four options distributed on a Likert scale from 0 to 3 (0 = not at all, 3 = severely) the presence or absence of anxiety symptoms in the past week, including the day of assessment. Clinically significant anxiety symptoms are determined with a cut-off score of 16 as reported in the reference literature (21).

#### 2.3.2 Highly sensitive person scale

The Highly Sensitive Person Scale (HSPS) is a 27-item self-report instrument designed to assess individual differences in Sensory Processing Sensitivity as a temperamental trait. Items are rated on a 7-point Likert scale (1 = ‘not at all’ to 7 = ‘extremely’), and summed to yield a total score (range 27–189), with higher scores indicating greater sensitivity to internal and external stimuli. The adaptation to Spanish has demonstrated adequate reliability and construct validity, allowing for interpretation in our samples (10). In line with the local adaptation, participants were classified as highly sensitive when the total score was ≥167 for women and ≥160 for men. The distribution of scores by sex was documented, and the HSPS variable was used both continuously (z-score) and categorically (HS/non-HS) in secondary analyses.

#### 2.3.3 Heart rate variability

Heart rate variability (HRV) was assessed from R–R interval recordings acquired with the Polar Team2 system (Polar^®^). Participants rested supine; R–R intervals were recorded continuously during the last 10 min of rest, from which an artifact-free 5-min segment was analyzed (17, 18). Processing was performed in Kubios HRV^®^ software (Kuopio, Finland). We applied automatic artifact correction (Low filter, cubic interpolation) and excluded segments with >5% corrected beats; when corrections were ≤5%, the segment was retained and the corrected-beat percentage was recorded. Detrending used Kubios smoothness-priors/polynomial detrending (order 3; default λ for 5-min data). Time-domain measures included RMSSD (ms), an index of vagal modulation (19) and SDNN (ms) overall variability reflecting combined autonomic influences (21, 22). We additionally computed PNS and SNS indices and Baevsky’s Stress Index (SI) as implemented in Kubios: PNS derives from mean R–R, RMSSD and Poincaré SD1 (linked to RMSSD), whereas SNS derives from mean R–R, SI and Poincaré SD2 (related to SDNN); indices are expressed as normalized deviations from population means (21, 23–25). For frequency-domain analysis, power spectra were estimated via the Welch periodogram (Hanning window, 50% overlap, resampled at 4 Hz), integrating standard bands VLF 0.0033–0.04 Hz, LF 0.04–0.15 Hz, HF 0.15–0.40 Hz. We report absolute power (ms²) and log-transformed values when needed. Respiration was spontaneous; respiratory rate (and/or HF peak) was included as a covariate, and we verified that the HF peak frequency fell within 0.15–0.40 Hz; sensitivity analyses excluded observations outside that range.

### 2.4 Procedure

Measurements were performed at the University of Magallanes Teaching and Research Center (CADI-UMAG) in Punta Arenas, where a structured history and medical record review were initially conducted. The assessment protocol consisted of the HSPS and BAI. Cardiac parameters were subsequently measured. HRV assessment included a resting baseline recording (a 5-minute artifact-free segment extracted from a 10-minute continuous recording) and a 5-minute post-exercise recording, also selected for analysis. The functional test consisted of the “step-up/knee raise” subtest of the Senior Fitness Test: 2 minutes of repetitions of knee raises to the specified angle (≥70°), with the number of valid executions recorded per participant. Before measurements, participants were asked to wear comfortable clothing and avoid caffeine, tobacco, alcohol, and strenuous exercise in the preceding 12 hours.

### 2.5 Statistical analysis

For this study we have chosen a Bayesian framework because it allows us to directly quantify evidence of both the presence and absence of effects (through Bayesian factors and ROPE-based decisions), which is particularly relevant in a relatively small sample where null findings are theoretically informative.

The effects of SPS on anxiety symptoms were assessed using a univariate Bayesian hierarchical generalized linear model (26) A similar multivariate model was then fitted, estimating the effect of anxiety symptoms on heart rate variability (HRV) indices in a single model.

For this last model, a second version was constructed that additionally considered the effect of High Sensory Processing on the overall effect. This approach allowed us to evaluate the mediating effect of sensory processing of anxiety symptoms on cardiac autonomic modulation.

Given that two measurements of the same variables were available for each individual, the model was adjusted to control for the influence of seasonality on the observed main effects. This was achieved by accounting for the within-subject correlation between measurements and adding a random intercept for each participant. This provided a robust estimate of the model parameters and the main effects of the study.

To improve the exploration of the parameter space, both the response variable and the predictor variables were standardized, centering them at 0 and adjusting them to the same scale. This transformation allowed for a clearer interpretation of the effect sizes, expressed in standard deviation units, facilitating comparisons between the effects of the different predictor variables.

For the linear coefficients, priors were chosen with a regularizing effect for the main linear effects and the standard deviation of the intercept per subject. This limits the effect of influential observations and contributes to model convergence.

Following the Sequential Effect Existence and Significance Testing (SEXIT) framework for describing parameter effects in Bayesian models (21), median and 95% credible intervals (CI95%) with high-density interval were reported as measures of centrality and uncertainty.

The probability of direction (pd) was used as a measure of the existence of an effect.

The proportion of the posterior distribution that falls outside the range of practical equivalence (ROPE) on the sign side was used as a measure of practical significance (ps), estimated at 0.1 standard deviations.

As a measure of absolute evidence for or against the null hypothesis, the Bayes Factor (BF10) was used using the Savage-Dickey density ratio against the point null hypothesis, evaluating whether this value has become more or less likely given the observed data (21).

For the interpretation of the Bayes Factor (BF), the following were considered: BF = 1: No evidence; 1 < BF ≤ 3: Anecdotal evidence; 3 < BF ≤ 10: Moderate evidence; 10 < BF ≤ 30: Strong evidence; 30 < BF ≤ 100: Very strong evidence; BF > 100: Extreme evidence (21).

For the proportion of the posterior distribution in the ROPE, the following are considered: <1%: Significant; <2.5%: Likely significant; ≤97.5% and ≥2.5%: Indeterminate significance; >97.5%: Likely insignificant; >99%: Insignificant (21).

All statistical analyses were calculated and implemented using the R statistical language (v4.5.0).

## 3 Results

### 3.1 High sensitivity and anxiety

**Figure 1:**
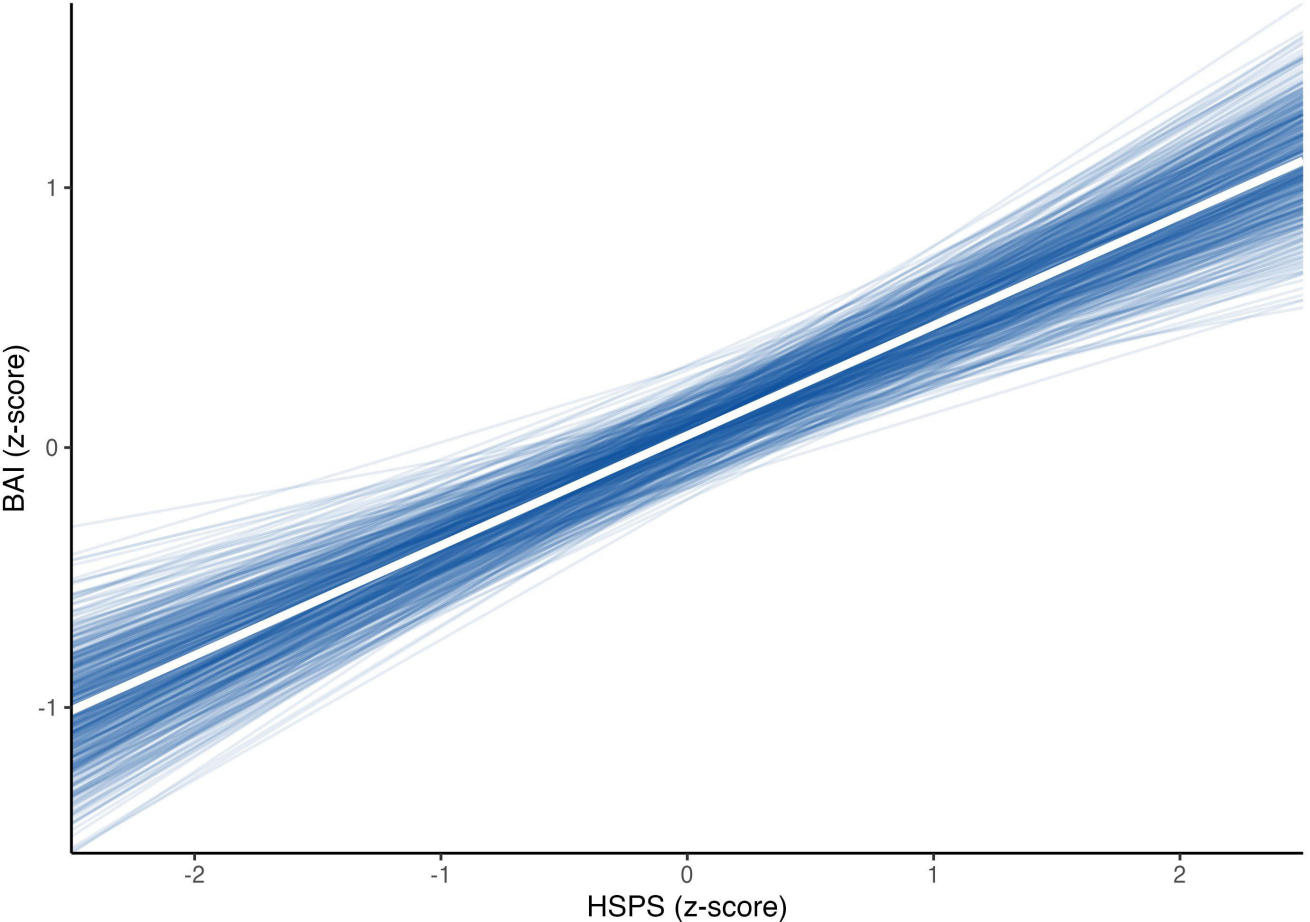
Effect of HSPS on BAI scores, adjusted for the effect of seasonality and intra-subject correlations.

Initially, it was observed that the HSPS (27) score was associated with an increase in the BAI score, showing that for everyone standard deviation increases in the HSPS, a proportional increase of 0.422 standard deviations in BAI scores could be expected. This corresponds to a moderate standardized association, suggesting that higher SPS is meaningfully related to higher anxiety symptoms in this sample (**Figure 1**)

On the other hand, the effect exerted by seasonality attributed to the study design provided evidence in favor of the null effect (BF = 0.08), suggesting that seasonality did not influence the modification of this relationship between HSPS and BAI.

### 3.2 Anxiety on HRV parameters

When evaluating the influence of BAI scores on the different HRV measures, we observed the following: in the time domains, we found evidence in favor of a null effect, suggesting that BAI scores did not influence either the RMSSD (BF = 0.046) or the SDNN (BF = 0.200). Regarding the frequency domains, we observed evidence of a null effect, similar to the findings in the time domains, for HF (BF = 0.070), LF (BF = 0.032), and VFL (BF = 0.038) (28) (see **Table 2**).

**Table 2:** Effect of BAI and HSPS scores on HRV response to exercise, adjusted for the effect of seasonality and intra-subject correlations. RMSSD (Root mean squared of successive differences between N-N intervals); SDNN (Standard Deviation of NN Intervals) HF (High Frequency); LF (Low Frequency); VLF (Very Low Frequency); PNSN (Parasympathetic Nervous System Index); SNSN (Sympathetic Nervous System Index).

PARÁMETRO		MEDIANA	BAJO	ALTO	PD	PS	ROPE	BF
RMSSD DELTA	BAI (Score)	0.003	-0.013	0.020	0.661	0.000	1.000	0.003
	HSPS (Score)	-0.013	-0.029	0.003	0.943	0.000	1.000	0.010
SDNN DELTA	BAI (Score)	-0.099	-0.262	0.78	0.868	0.496	0.504	0.056
	HSPSS (Score)	-0.045	-0.212	0.127	0.697	0.262	0.726	0.033
HF DELTA	BAI (Score)	0.043	-0.126	0.221	0.684	0.257	0.727	0.033
	HSPSS (Score)	-0.104	-0.278	0.068	0.880	0.518	0.481	0.059
LF DELTA	BAI (Score)	-0.107	-0.275	0.077	0.881	0.530	0.468	0.063
	HSPSS (Score)	0.072	-0.101	0.242	0.790	0.374	0.632	0.041
VLF DELTA	BAI (Score)	-0.118	-0.301	0.058	0.905	0.580	0.416	0.071
	HSPS (Score)	0.061	-0.119	0.243	0.749	0.335	0.658	0.038
PNSN DELTA	BAI (Score)	0.039	-0.143	0.198	0.673	0.239	0.741	0.032
	HSPS (Score)	0.024	-0.151	0.188	0.602	0.189	0.770	0.030
SNSN DELTA	BAI (Score)	0.012	-0.027	0.053	0.0721	0.000	1.000	0.008
	HSPS (Score)	0.010	-0.028	0.049	0.700	0.000	1.000	0.008

RMSSD (Root mean squared of successive differences between N-N intervals); SDNN (Standard Deviation of NN Intervals) HF (High Frequency); LF (Low Frequency); VLF (Very Low Frequency); PNSN (Parasympathetic Nervous System Index); SNSN (Sympathetic Nervous System Index).

### 3.3 High sensitivity in the interaction between anxiety and heart rate variability

Examining the effect of the HSPS on the relationship between BAI and HRV, we observed that, after considering the marginal effect of the high sensitivity profile on the influence of BAI scores, the aforementioned effects were non-significant. (See **Table 1**).

### 3.4 HSPS and BAI on the HRV response to exercise

**Figure 2:**
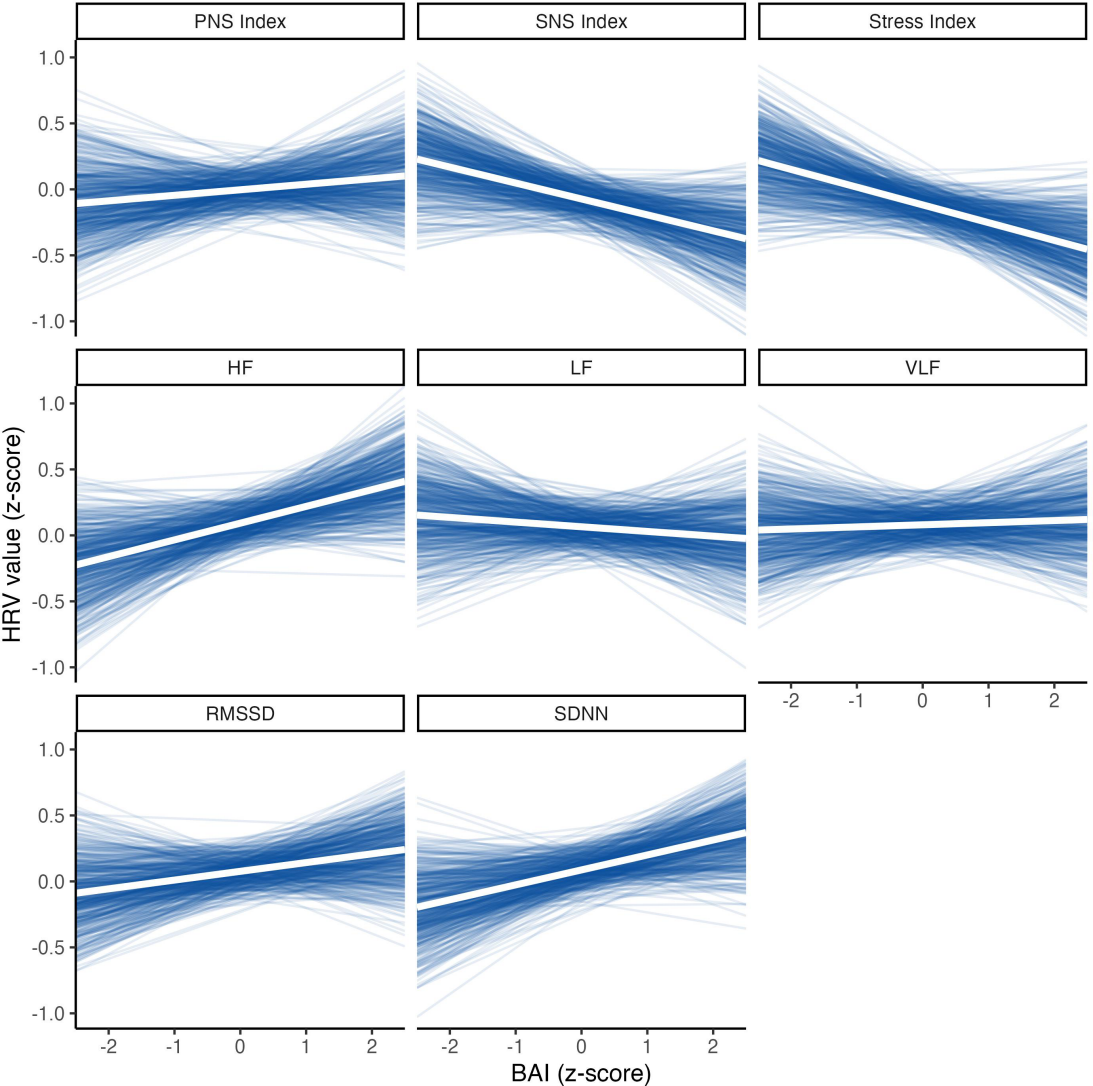
Effect of BAI scores on HRV measurements, adjusted for the effect of seasonality and intra-subject correlations.

When examining the effect of HSPS and BAI on the HRV response to exercise, none of the HSPS or BAI scores had a significant effect on any of the HRV domains in response to exercise (**Figure 2**).

## 4 Discussion

In this high-southern community sample, SPS was consistently associated with greater anxiety, whereas neither resting HRV nor its response to brief exercise showed clinically relevant associations with anxiety, nor evidence of moderation by SPS. Taken together, the findings suggest that, in older individuals from the far south, SPS operates primarily as a psychological vulnerability, rather than as an autonomic dysfunction detectable with 5-minute HRV recordings. Our findings can be interpreted within the broader framework of environmental sensitivity. In this view, Sensory Processing Sensitivity (SPS) does not function solely as a vulnerability factor, but rather as a marker of differential susceptibility to environmental influences. According to this perspective, individuals high in SPS may experience stronger negative outcomes under stressful or unsupportive conditions, yet they can also benefit more profoundly from positive and nurturing environments (7).

In this context, the significant relationship found between high sensory processing sensitivity (SPS) and elevated anxiety levels is consistent with previous studies, which have shown that individuals with high SPS often experience greater emotional reactivity and are more easily overstimulated (4). These traits are frequently accompanied by higher levels of anxiety.

From this perspective, SPS could be understood as a psychological vulnerability factor, particularly in situations that are emotionally intense or stressful. This may be especially true for older adults living in sub-Antarctic regions, where environmental factors such as seasonal changes have been shown to impact psychological well-being (29). Additionally, this invites new studies focused on earlier stages of the life cycle of older people who possess the trait of high sensitivity (11), since its presence during periods of childhood could be a predictive factor for the development of anxiety disorders during adulthood. For instance, Jagiellowicz et al. (2016) demonstrated that high-SPS individuals respond more intensely to both positive and negative emotional stimuli, particularly when early life environments were supportive. Similarly, Lionetti et al. (2018) identified distinct sensitivity groups—low-, medium-, and high-sensitive individuals—showing that those with higher sensitivity display greater emotional responsiveness and neuroticism, as well as lower extraversion. Together, these findings align with the present results, reinforcing the view that SPS primarily manifests through heightened emotional and psychological responsiveness, even in the absence of measurable physiological dysregulation (30, 31).

In contrast, no significant relationships were observed between the trait of high sensitivity and heart rate variability indices, nor were moderating effects of SPS found on the relationship between anxiety and HRV, a finding that contrasts with the initial hypothesis. However, several studies have suggested that HRV does not always adequately reflect the presence of anxiety symptoms, especially when taken at rest, and may be related to multiple factors such as comorbidity with other diagnoses or the activation of anxiety during the measurements performed (32). However, after performing cardiac measurements after exercise, the results did not generate major changes, which, in line with what was indicated, could be associated with the absence of anxiety symptoms during the evaluation (33).

In light of the above, it is possible to suggest that one explanation for the absence of an association between SPS and HRV could be related to the presence of compensatory mechanisms in individuals that allow for emotional regulation, for example, it has been observed in the literature that the more the subject focuses on external effects of behavior, such as movements, the better their motor control performance and the less effect perceived at the HRV level, unlike those who focus on their own body and are aware of the sensations produced by anxiety, thus altering measurements such as HRV (34). This is why some highly sensitive individuals tend to develop adaptive strategies in the face of overstimulation and emotional reactivity, focusing in aspects like performance, thoughts of self-compassion or emotional acceptance that in the perception of one’s own body.

These strategies could mediate the autonomic nervous system in the presence of anxiety symptoms, achieving a more adaptive response that does not generate alterations in the autonomic system (35). In this sense, emotional self-regulation could modulate the physiological expression of psychological distress, maintaining stable HRV parameters.

Overall, the results indicate that sensory processing sensitivity did not exert a significant modulating effect on the HRV response to physical exercise. This could suggest that the influence of this personality trait manifests itself primarily psychologically and not necessarily as a physiological response, reinforcing the idea that compensatory and self-regulation mechanisms could mediate alterations in HRV in the presence of anxiety symptoms in highly sensitive individuals. Likewise, it is possible to assume that the physiological thresholds of highly sensitive individuals are higher, requiring higher levels of anxiety and stimulation to obtain more immediate autonomic responses. Furthermore, it is necessary to consider other variables in the study, such as the presence of comorbidities and medication of the participants; these factors could influence the results associated with HRV.

These results are important for understanding the interactions between physiological and psychological factors in older adults with the trait of high sensitivity to sensory processing in the city of Punta Arenas, specifically between the variables studied, namely, anxiety and heart rate variability, in this manner the results of this study, which confirm the relationship between High Sensory Processing Sensitivity (SPS) and anxiety, are highly relevant in the clinical setting. The implications of these findings are favorable, as they enable the development and implementation of cognitive-behavioral psychological strategies (therapeutic or performance-based) aimed at the population with SPS. The goal of these interventions is to improve the subject’s ability, particularly older adults, to cope with and manage environmental stressors in a more adaptive way. This line of action is crucial, as it aligns with the study’s suggestion that emotional self-regulation mechanisms are key to modulating the anxiety response and preventing its manifestation at the physiological level.

On the other hand, one of the limitations observed in the study relates to the representativeness of the sample, as the selection of 101 participants limits the generalizability of the findings to the general population. Furthermore, future research needs to consider new variables, such as comorbidities that may bias physiological measurements and emotional self-regulation strategies that allow for a better understanding of the processes that modulate the relationship between SPS, anxiety, and HRV.

## 5 Conclusions

In conclusion, although this study provided evidence for a positive association between Sensory processing sensitivity (SPS) and anxiety levels in older adults in the city of Punta Arenas. This finding is particularly relevant, as it highlights the importance of considering high sensitivity as a psychological vulnerability factor in old age, a stage in which adaptive resources and support networks play a crucial role.

Furthermore, the results contribute to broadening our understanding of individual differences in emotional experience and how these impact the mental well-being of older adults in southern settings and those far from large urban centers. In the future, it is necessary to further explore the relationship between SPS, physiological indicators such as HRV, and psychological health through longitudinal studies with larger samples. Such research could guide preventive interventions and psychosocial support programs that promote emotional regulation and adaptive coping, contributing to both clinical and public mental health policies for the increasingly aging populations.

## Data Availability

The raw data supporting the conclusions of this article will be made available by the authors, without undue reservation.

## References

[1] Mabe-Castro D Gomez KT Castillo-Aguilar M Jannas-Vela S Guzmán-Muñoz E Valdés-Badilla P . Frailty, seasonal sensitivity and health-related quality of life in older people living in high southern latitudes: a Bayesian analysis. Can Geriatr J. (2024) 27:56–62. doi: 10.5770/cgj.27.719, PMID: 38433882 PMC10896209

[2] Castillo-Aguilar M Mabe-Castro D Mabe-Castro M Mendes TT Concha-Cisternas Y Guzmán-Muñoz E . The winter is coming: seasonal variations in BDNF levels among older adults in a high-latitude region — a preliminary study. Front Psychiatry. (2025) 16:1692566. doi: 10.3389/fpsyt.2025.1692566, PMID: 41164095 PMC12560003

[3] Alvarado-Aravena C Estrada-Goic C Núñez-Espinosa C . Sintomatología depresiva y calidad de vida en estudiantes de medicina en alta latitud sur. Rev Med Chile. (2021) 149:357–65. doi: 10.4067/s0034-98872021000300357, PMID: 34479314

[4] Acevedo B Aron E Pospos S Jessen D . The functional highly sensitive brain: A review of the brain circuits underlying sensory processing sensitivity and seemingly related disorders. Philos Trans R Soc Lond B Biol Sci. (2018) 373:20170161. doi: 10.1098/rstb.2017.0161, PMID: 29483346 PMC5832686

[5] Kenwood MM Klain NH Barbas H . The prefrontal cortex, pathological anxiety, and anxiety disorders. Neuropsychopharmacology. (2022) 47:260–75. doi: 10.1038/s41386-021-01109-z, PMID: 34400783 PMC8617307

[6] Pluess M . Individual differences in environmental sensitivity. Child Dev Perspect. (2015) 9:138–43. doi: 10.1111/cdep.12120

[7] Greven CU Lionetti F Booth C Aron EN Fox E Schendan HE . Sensory processing sensitivity in the context of environmental sensitivity: A critical review and development of research agenda. Neurosci Biobehav Rev. (2019) 98:287–305. doi: 10.1016/j.neubiorev.2019.01.009, PMID: 30639671

[8] Huirimilla Casanova L Henríquez Luhr J Castillo-Aguilar M Chacón A Pérez-Chacón M Harris King K . La Alta Sensibilidad del Procesamiento Sensorial y su relación con el equilibrio postural en personas mayores. Retos. (2024) 58:308–14. doi: 10.47197/retos.v58.106788

[9] Schaefer M Kühnel A Gärtner M . Sensory processing sensitivity and somatosensory brain activation when feeling touch. Sci Rep. (2022) 12:12024. doi: 10.1038/s41598-022-15497-9, PMID: 35835782 PMC9283459

[10] Chacón A Pérez-Chacón M Borda-Mas M Avargues-Navarro ML López-Jiménez AM . Cross-cultural adaptation and validation of the Highly Sensitive Person Scale to the adult Spanish population (HSPS-S). Psychol Res Behav Manage. (2021) 14:1041–52. doi: 10.2147/PRBM.S321277, PMID: 34285606 PMC8286783

[11] McMahon K Anand D Morris-Jones M Rosenthal MZ . A path from childhood sensory processing disorder to anxiety disorders: The mediating role of emotion dysregulation and adult sensory processing disorder symptoms. Front Integr Neurosci. (2019) 13:22. doi: 10.3389/fnint.2019.00022, PMID: 31338029 PMC6629761

[12] Park J Moghaddam B . Impact of anxiety on prefrontal cortex encoding of cognitive flexibility. Neuroscience. (2017) 345:193–202. doi: 10.1016/j.neuroscience.2016.06.013, PMID: 27316551 PMC5159328

[13] Waters AM Craske MG . Towards a cognitive-learning formulation of youth anxiety: A narrative review of theory and evidence and implications for treatment. Clin Psychol Rev. (2016) 50:50–66. doi: 10.1016/j.cpr.2016.09.008, PMID: 27693665

[14] Schaaf RC Miller LJ Seawell D O’Keefe S . Children with disturbances in sensory processing: a pilot study examining the role of the parasympathetic nervous system. Am J Occup Ther. (2003) 57:442–9. doi: 10.5014/ajot.57.4.442, PMID: 12911086

[15] Pham T Lau ZJ Chen SHA Makowski D . Heart rate variability in psychology: A review of HRV indices and an analysis tutorial. Sensors (Basel). (2021) 21:3998. doi: 10.3390/s21123998, PMID: 34207927 PMC8230044

[16] Cervin M . Sensory processing difficulties in children and adolescents with obsessive-compulsive and anxiety disorders. Res Child Adolesc Psychopathol. (2023) 51:223–32. doi: 10.1007/s10802-022-00962-w, PMID: 36149521 PMC9867656

[17] Veloza L Acevedo C Pérez E . Análisis de la variabilidad de la frecuencia cardiaca y su aplicación en la valoración del sistema nervioso autónomo. Rev Cienc Salud. (2019) 17:75–89. *(agregada para sustentar datos fisiológicos y ecuaciones RR)*. doi: 10.1016/j.rccar.2019.01.006

[18] Castillo-Aguilar M Valdés-Badilla P Herrera-Valenzuela T Guzmán-Muñoz E Delgado-Floody P Cristóbal Andrade D . Cardiac autonomic modulation in response to muscle fatigue and sex differences during consecutive competition periods in young swimmers: A longitudinal study. Front Physiol. (2021) 12:769085. doi: 10.3389/fphys.2021.769085, PMID: 34867474 PMC8637437

[19] Buchheit M Chivot A Parouty J Mercier D Al Haddad H Laursen PB . Monitoring endurance running performance using cardiac parasympathetic function. Eur J Appl Physiol. (2010) 108:1153–67. doi: 10.1007/s00421-009-1317-x, PMID: 20033207

[20] Castillo-Aguilar M Mabe-Castro D Medina D . Enhancing cardiovascular monitoring: a non-linear model for characterizing RR interval fluctuations in exercise and recovery. Sci Rep. (2025) 15:8628. doi: 10.1038/s41598-025-93654-6, PMID: 40074820 PMC11904009

[21] Berntson GG Bigger JT Jr Eckberg DL Grossman P Kaufmann PG Malik M . Heart rate variability: origins, methods, and interpretive caveats. Psychophysiology. (1997) 34:623–48. doi: 10.1111/j.1469-8986.1997.tb02140.x, PMID: 9401419

[22] Buchheit M Gindre C . Cardiac parasympathetic regulation: respective associations with cardiorespiratory fitness and training load. Am J Physiol Heart Circ Physiol. (2006) 291:H451–H458. doi: 10.1152/ajpheart.00008.2006, PMID: 16501030

[23] Acharya UR Joseph KP Kannathal N Lim CM Suri JS . Heart rate variability: a review. Med Biol Eng Comput. (2006) 44:1031–51. doi: 10.1007/s11517-006-0119-0, PMID: 17111118

[24] Baevsky RM . Methodical recommendations: use of KARDiVAR system for determination of the stress level and estimation of the body adaptability. Standards of measurements and physiological interpretation (2008). Available online at: https://api.semanticscholar.org/CorpusID:29215863 (Accessed October 27, 2025).

[25] Yoo HH Yune SJ Im SJ Kam BS Lee SY . Heart rate variability-measured stress and academic achievement in medical students. Med Princ Pract. (2020) 30:193–200. doi: 10.1159/000513781, PMID: 33326983 PMC8114035

[26] van de Schoot R Depaoli S King R Kramer B Märtens K Tadesse MG . Bayesian statistics and modelling. Nat Rev Methods Prim. (2021) 1:1. doi: 10.1038/s43586-020-00001-2

[27] Beck AT Epstein N Brown G Steer RA . An inventory for measuring clinical anxiety: psychometric properties. J Consult Clin Psychol. (1988) 56:893–7. doi: 10.1037/0022-006X.56.6.893, PMID: 3204199

[28] Makowski D BenShachar MS Chen SHA Lüdecke D . Indices of effect existence and significance in the bayesian framework. Front Psychol. (2019) 10:2767. doi: 10.3389/fpsyg.2019.02767, PMID: 31920819 PMC6914840

[29] Henríquez J Henríquez W MéndezMuñoz R Ibinarriaga T MabeCastro D MabeCastro M . Cognitive impairment and anxiety in older adults: characterizations in a high southern latitude population. RevInvestigInnovCiencSalud. (2025) 7:111. doi: 10.46634/riics.326

[30] Jagiellowicz J Aron A Aron EN . Relationship between the temperament trait of sensory processing sensitivity and emotional reactivity. Soc Behav Pers. (2016) 44:185–200. doi: 10.2224/sbp.2016.44.2.185

[31] Lionetti F Aron A Aron EN Burns GL Jagiellowicz J Pluess M . Dandelions, tulips and orchids: evidence for the existence of low-sensitive, medium-sensitive and high-sensitive individuals. Transl Psychiatry. (2018) 8:24. doi: 10.1038/s41398-017-0090-6, PMID: 29353876 PMC5802697

[32] Liao KH Sung CW Chu SF Chiu WT Chiang YH Hoffer B . Reduced power spectra of heart rate variability are correlated with anxiety in patients with mild traumatic brain injury. Psychiatry Res. (2016) 243:34956. doi: 10.1016/j.psychres.2016.07.001, PMID: 27449003

[33] Gourley B . Exploring the relationship between anxiety sensitivity and heart rate variability [Master’s thesis]. Eastern Michigan University, Ypsilanti (MI (2019). p. 123. Available online at: https://commons.emich.edu/theses/982 (Accessed October 27, 2025).

[34] Mullen R Faull A Jones ES Kingston K . Attentional focus and performance anxiety: effects on simulated race-driving performance and heart rate variability. Front Psychol. (2012) 3:426. doi: 10.3389/fpsyg.2012.00426, PMID: 23133431 PMC3488937

[35] Filho E Di Fronso S Mazzoni C Robazza C Bortoli L Bertollo M . My heart is racing! Psychophysiological dynamics of skilled racecar drivers. Psychol Sport Exerc. (2015) 16:132–41. doi: 10.1016/j.psychsport.2014.09.007, PMID: 25555177

